# Occurrence of Phthalate Esters in Coffee and Risk Assessment

**DOI:** 10.3390/foods12051106

**Published:** 2023-03-05

**Authors:** Salvatore Velotto, Jonathan Squillante, Agata Nolasco, Raffaele Romano, Teresa Cirillo, Francesco Esposito

**Affiliations:** 1 Department of Promotion of Human Sciences and the Quality of Life, University of Study of Roma “San Raffaele”, Via di Val Cannuta, 247, 00166 Roma, Italy; 2 Department of Agricultural Sciences, University of Naples Federico II, Via Università, 100, 80055 Portici, Italy; 3 Department of Public Health, University of Naples Federico II, Via Sergio Pansini, 5, 80131 Naples, Italy

**Keywords:** coffee, packaging, coffee machine, phthalic acid esters, risk assessment, coffee consumption

## Abstract

Coffee, one of the most widely consumed beverages in the world, is commercialized as powder and beans in different types of packaging and extracted through several methods. In this regard, the present study focused on evaluating the concentration of two of the most used phthalates in plastic materials (bis(2-ethylhexyl)phthalate (DEHP) and di-butyl phthalate (DBP)) in coffee powder and beverages to assess their migration from different packaging and machines. Furthermore, the levels of exposure to these endocrine disruptors in regular coffee consumers were estimated. Samples of packaged coffee powder/beans (*n* = 60) from different forms of packaging (multilayer bag, aluminum tin, and paper pod) and coffee beverages (*n* = 40) that were differently extracted (by professional espresso machine (PEM), Moka pot (MP), and home espresso machine (HEM)) were analyzed by extraction of the lipid fraction, purification, and determination by gas chromatography–mass spectrometry (GC/MS). Risk due to consumption of coffee (1–6 cups) was assessed based on tolerable daily intake (TDI) and incremental lifetime cancer risk (ILCR). No significant differences emerged in DBP and DEHP concentrations among different types of packaging (multilayer, aluminum, and paper), whereas higher levels of DEHP were reported in beverages extracted by PEM (6.65, 2.58–11.32) than by MP (0.78, 0.59–0.91) and HEM (0.83, 0.62–0.98). The presence of higher DEHP levels in coffee beverages than in coffee powder may be due to its leaching through machine components. However, the levels of PAEs did not exceed the specific migration limits (SMLs) set out for food contact materials (FCM), and exposure to PAEs from coffee beverages was low, justifying the small risk due of its consumption. Consequently, coffee can be considered a safe beverage for exposure to some phthalic acid esters (PAEs).

## 1. Introduction

Phthalates or phthalic acid esters (PAEs) are organic additives widely used in the plastic industry as plasticizers that provide flexibility, transparency, and durability to polymers. However, they are endocrine disruptors due to their capability to alter the grow and reproduction as well as induce neurotoxicity, genotoxicity, and carcinogenicity [1,2,3]. Bis(2-ethylhexyl) phthalate (DEHP) and dibutyl phthalate (DBP) are among the most toxic phthalates [4]. Furthermore, they are widely used in PVC products, medical devices, adhesives, paints, and printing inks and are also the most abundant PAEs in soil due to atmospheric deposition [5]. Under the specific conditions of temperature, salinity, pH, and radiation, PAEs may be released in the environment because they are not chemically bound to the matrix [6,7,8]. As a result, food and beverages, which represent the main source of human exposure, are likely to contain significant amounts of these plasticizers [9]. According to Net et al., diet may contribute more than 67% of human exposure [10]. The relevant concentrations of PAEs in foods and beverages could be a consequence of their accumulation along the food production chain. In particular, the processing chain of coffee consists of several steps, such as harvest, processing, packing, and extraction, that could lead to PAEs migration from the environment, packaging, and machine materials.

For these reasons, the present study focused on the potential contamination of coffee, it being one of the most marked and consumed beverages worldwide. According to European Coffee Report 2018/2019 [11], Italy recorded the highest import change (+33%), highlighting the relevant consumption of this beverage, which stands at 1–3 cups of coffee per day for 70% of consumers and 4–6 cups per day for the remaining 30%, according to Lanfranchi et al. [12].

Over the last few decades, several European regulations have been implemented because of the increasing focus on consumer food safety due to high exposure to phthalates. In particular, the use of food contact materials (FCM) must adhere to strict requisites according to Regulation (EC) No 1935/2004 [13], and specific migration limits (SML) for individual substances (such as bis(2-ethylhexyl)phthalate (DEHP) and di-butyl phthalate (DBP)) based on a toxicological assessment laid down by Commission Regulation (EU) No 10/2011 [14]. The most-used packaging materials in coffee industry are paper, aluminum, plastic, and a mix of them in a multilayer (typically 75% paper, 5% aluminum, and 20% low-density polyethylene (LDPE), based on the type of product (e.g., beans and their powder, pods, or capsule). Some products can also have double packaging, such as pods (paper and multilayer) or capsules (plastic and multilayer). On the other hand, coffee machines (e.g., espresso machines, Moka pots, kettles) differ significantly in extraction methods, equipment and materials, such as steel, aluminum, Teflon, and silicone-based materials; they also differ on cost, convenience, and the type of brewed coffee that can be obtained. Previous studies assessed the occurrence of PAEs in coffee, showing different levels of them depending on the state, packaging, and/or extraction method [15,16,17,18]. Herein, the occurrence of DEHP and DBP in both coffee powder and beverages is discussed to investigate the potential contamination of foodstuffs at multiple stages. Different packaging (multilayer, paper, tin) and machines (Moka pots (MP)), pod home espresso machines (HEM), and professional espresso machines (PEM)) were taken into account and compared with each other. Secondly, an assessment of risk based on the consumption of 1–6 cups/day was estimated through a deterministic approach.

## 2. Materials and Methods

### 2.1. Sampling

Sampling was carried out on 60 differently packaged coffee powders/beans: 15 samples of coffee powder vacuum-packed in a multilayer bag, and 15 samples of beans packed in a multilayer bag, freshly ground (Multilayer), 15 samples of coffee paper pods in a multilayer bag (Pod), and 15 samples of coffee powder packaged in an aluminum tin (Aluminium). The samples were purchased from different markets in Naples. Three aliquots of powder were collected from the packaging, whereas the analysis was performed in duplicate, both times on 10 g of coffee powder.

Regarding coffee machines, the following products were taken into account: multilayer package (*n* = 10) and aluminum tin (*n* = 10) (for MP-brewed coffee), multilayer bag (*n* = 10) (for PEM-brewed coffee), and coffee pods (*n* = 10) (for HEM-brewed beverages) were taken into account. MP and HEM underwent a wash-out phase of 10 g of coffee. An aliquot of 12 g of coffee powder was used to extract the beverages by the matched machine using Milli-Q water. The liquid sample was frozen and freeze-dried. The analysis was performed in duplicate on 30 mL of coffee beverage.

### 2.2. Chemical and Reagents

The standards for DEHP and DBP were purchased from Sigma-Aldrich (Shneldorf, Germany). Acetonitrile, *n*-hexane, and anhydrous sodium sulfate were purchased from Merck & Company, Inc. (Kenilwortf, NJ, USA). Florisil (60/100 mesh) was purchased from Supelco Bellefonte, PA, USA and Bondesil (PSA 40 UM) from Varian Palo Alto, CA, USA. All the reagents used in the experiment were of the highest grade available.

### 2.3. Instrumentation

The analysis of DEHP and DBP was performed using a gas chromatography-mass spectroscopy (GC-MS, Agilent 7890A GC system coupled to an Agilent 5975C mass selective detector (MSD) (Agilent Technologies, Santa Clara, CA, USA)). The method imposed an injector of temperature 260 °C, a detector temperature of 310 °C and an initial oven temperature of 100 °C (holding time 1 min). After the injection, the ramp rate was programmed from 100 °C to 280 °C at 15 °C/min, maintaining this temperature for 10 min. The transfer line of the GC-MS interface was held at 280 °C. Samples (1 μL) were injected into the capillary gas chromatography column in a splitless mode. The carrier gas was high-purity helium (99.999%) at a 1.0 mL/min flow rate.

The acquisition was performed in Selected Ion Monitoring (SIM) using 3 ions per compound: m/z 149, 167, and 279 for DEHP and 149, 205, and 223 for DBP. The retention times were 21.8 and 17.2 min for DEHP and DBP, respectively.

### 2.4. Calibration Curves

Calibration curves were performed by diluting the DEHP and DBP standards in n-hexane at concentrations from 1 to 100 ng/mL. The PAEs concentrations in the samples were determined by comparing the corresponding peak areas with the calibration curve. The linearity obtained from regression analysis for both chemicals showed regression coefficients (R^2^) > 0.99. The pure solvent was analyzed as blank sample for each batch, and the average concentration value was subtracted from the DEHP and DBP detection values. To reduce the instrumental background due to ubiquitous contamination, a blank run was performed after every six determinations. In addition, the solvents used to wash the syringe were changed frequently.

### 2.5. Phthalic Acid Esters (PAEs) Extraction and Clean-Up

Extraction and purification of PAEs were performed according to Tsumura et al. [19], with minor modifications [20]. The tools were preliminarily heated in muffle at 400 °C and washed with acetone and n-hexane. No plastic equipment was used during the analysis. An aliquot of 10 g of coffee powder was taken from the three types of packaging considered, mixed with 10 mL of acetonitrile in a centrifuge tube and shaken manually for 1 min. Instead, the coffee beverage (30 g) was dried using Rotavapor at 45 °C, and then it followed the same procedure as the powder. The sample was subjected to an ultrasonic bath for 10 min and centrifuged at 515 RCF for 10 min. The supernatant was collected in a separatory funnel and the process was repeated. A volume of 10 mL of n-hexane saturated with acetonitrile were added to the separator funnel and shaken for 5 min. The acetonitrile phase containing the PAEs was transferred to a flask and dried using Rotavapor at 45 °C (BÜCHI Labortechnik, Switzerland). Then, the sample was mixed with 5 mL of n-hexane and the solution underwent cleaning as described below.

The purification process was carried out on a custom-packed chromatographic column composited by wadding (placed in an oven at 100 °C for 1 h), 2 g of Florisil activated for 2 h at 200 °C, 0.5 g of Bondesil and 1 g of anhydrous sodium sulfate (Na_2_SO_4_). Then, 10 mL of acetone and hexane was added to the column and partially eluted. The sample was loaded and eluted onto the column. A solution of 10 mL acetone: n-hexane (3:2) was added to the column. Then, the eluate was collected and dried by Rotavapor at 40 °C. The sample was then transferred with 1 mL of n-hexane in a vial and injected into the GC/MS.

Three samples of each category (in triplicate) were spiked with standard solutions at concentrations 0.5, 1.0, and 2.0 μg/mL for DEHP and 0.5, 1.0, and 2.0 μg/mL for DBP; they were then processed as coffee samples. Recoveries were 98 ± 10% for DEHP and 98 ± 9% for DBP.

### 2.6. Risk Assessment

The potential risk of coffee consumption was assessed based on tolerable daily intake (TDI) for DEHP and DBP and incremental lifetime cancer risk (ILCR) for DEHP.

Estimated daily intake (*EDI*, ng/kg_bw_/day) was previously calculated according to the following formula [22]:

$$EDI=\frac{IR\times C}{BW}$$

- *C*: Median concentrations of PAE (ng/g)
- *IR*: Intake rate of coffee (g/day) set to 1–6 coffee (30 mL/coffee) [12]
- *BW*: Body weight (kg_bw_) for toddlers (11.3), adolescents (52.6), and adults (69.7) [22]

Non-carcinogenic risk was evaluated through the comparison of *EDI* and *TDI* of DEHP (50 μg/kg_bw_/day) and DBP (10 μg/kg_bw_/day).

Carcinogenic risk was assessed through the following equation [21]:

$$ILCR=\frac{EDI\times EF\times TE\times SF}{AT}$$

- *EF*: Exposure frequency to the contaminant (350 days/year)
- *TE*: Total exposure (70 year)
- *AT*: Average lifetime time for non-carcinogenic risk (*TE* × 365 days/year)
- *SF*: Slope factor (μg/kg_bw_/day)*^−^*^1^.

According to USEPA, the slope factor of DEHP is 14 (μg/kg_bw_/day)^−1^. USEPA considers an *ILCR* (dimensionless) between 1 × 10^−4^ and 1 × 10^−6^ as an acceptable range for the risk of developing cancer over a human lifetime, whereas values > 1 × 10^−4^ are considered an unacceptable risk [21]. Instead, Health Canada proposes a lower threshold of 1 × 10^−5^ for the risk of developing cancer [23].

### 2.7. Statistical Analysis

The distribution of the data was evaluated with a Shapiro–Wilk test. A one-way ANOVA analysis using SPSS 20.0 software (IBM Corp, Armonk, NY, USA) was performed to assess differences between DEHP and DBP concentrations in powdered and liquid coffee. Significance was set at *p* < 0.05.

According to Fasano et al. the limits of detection (LOD) and limits of quantification (LOQ) were expressed as the mean blank plus three blank standard deviations, and the LOQ was expressed as three times the LOD. The LOD were 0.300 ng/mL for DEHP and 0.800 ng/mL for DBP, respectively [24]. Concentrations below the LOQ were assumed equal to the LOD, whereas concentrations below the LOD were considered equal to 0 (lower-bound approach).

## 3. Results and Discussion

### 3.1. PAEs Concentrations in Coffee Powder and Coffee Beverage

The median values of DBP and DEHP concentrations in the coffee powder samples contained in different forms of packaging are shown in Table 1. DEHP showed higher values than DBP for the three types of packaging considered. The highest DBP values were recorded for aluminum packs (0.58 ng/g), whereas DEHP reported higher levels in multilayer packs (5.01 ng/g). However, the analysis of the coffee powder showed no significant differences among the packaging materials used (multilayer, aluminum, and pods).

It should be noted, however, that the levels of PAEs in the coffee beverages were affected by the type of machine used. The values showed higher DEHP concentrations in coffee extracted by PEM (6.65 ng/g), whereas DBP concentrations were similar among coffee extracted by the three different coffee machines (Table 2).

The data highlighted that regardless of packaging and coffee machines, DEHP was the most abundant PAE in coffee powder and beverages. The contamination of the coffee powder, given the lack of significant differences, could be attributable to environmental contamination during the production process rather than related to a migration phenomenon in the packaging materials. In pre-packed steps (e.g., transportation, storage, roasting, grinding), PAEs could be released due to materials and environmental conditions. PAE levels in coffee powder were also analyzed by Guo et al. [25], who reported no occurrence of DEHP and higher levels of DBP (14.4 ng/g) than ours.

Likewise, Di Bella et al. [18] showed lower values for DEHP (2.09 ± 0.35 ng/g) and higher values for DBP (3.45 ± 0.82 ng/g) in coffee powder from pods, whereas they observed an opposite trend in coffee powder for MP (DEHP: 32.85 ± 1.89 ng/g; DBP: <LOQ). In coffee beverages, they found higher values of plasticizers in the beverage extracted by an HEM (DEHP: 3.89 ± 2.14 ng/mL; DBP: 3.40 ± 1.24 ng/mL). Similarly to our study, the levels of DEHP were highly variable but higher (from 8.63 to 52.52 ng/mL) than those of DBP (<LOQ) in the espresso extracted with MP.

On the other hand, in the present study, the coffee beverages obtained by MP and HEM showed lower DEHP levels compared to PEM.

However, since a coffee (30 mL) provided more DEHP than its proportion of powder (12 g), the migration of the plasticizer could depend on the materials of PEM (e.g., seal, pipe) and their wear degree [18]. In addition, the higher contact surface and pressure (10 bar in PEM and 1.5 bar in MP) could also affect the migration of DEHP, since these two parameters impact on the extraction of chemicals [26]. Overall, the PAEs in beverages could be leaked by machine rather than powder. However, overlooking the wear degree of PEM was a limitation with regard to differences among machines.

According to Commission Regulation (EU) No 10/2011, SMLs were applied for substances used as additives and polymers FCM [14]. Values of 1.5 mg/kg and 0.3 mg/kg were set for DEHP and DBP, respectively. From the data provided above, it can be concluded that coffee powders and espressos did not exceed the SMLs for either of these PAEs. In this respect, packaging and machines seem to fall within the requirements for the manufacturing and marketing of plastic materials and products.

### 3.2. Risk Assessment

**Figure 1:**
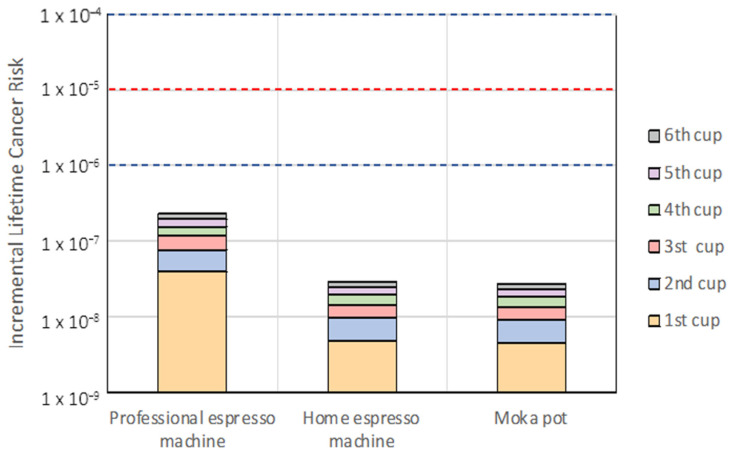
Incremental lifetime cancer risk (ILCR) in subjects consuming 1–6 cups of coffee extracted through different machines. The blue (1 × 10^−6^ and 1 × 10^−4^) and red (1 × 10^−5^) dashed y-intercept is the safety threshold as described in the study.

The risk assessment of exposure to PAEs through coffee intake was carried out on a daily coffee consumption of 1 to 6 cups (30 mL/cup) per day, according to Lanfranchi et al. [9]. The results showed no potential non-carcinogenic risk based on *EDI*. The exposure to DEHP and DBP through consumption of each type of coffee was well below the TDI. The values ranged from 0.33 ng/kg_bw_/day (for one cup of coffee from MP) to 17.17 ng/kg_bw_/day (for six cups of coffee from a PEM) for DEHP, whereas the values ranged from 0.030 ng/kg_bw_/day (for one cup of coffee from PEM) to 0.31 ng/kg_bw_/day (for six cups of coffee from HEM) for DBP. Likewise, the potential carcinogenic risk of DEHP did not exceed threshold values of 1 × 10^−5^ or 1 × 10^−4^ (Figure 1). The highest values were 2.31 × 10^−7^ for the consumption of six cups of coffee extracted by PEM. Therefore, the reasonable consumption of coffee beverages does not raise concerns regarding DEHP and DBP exposure.

## 4. Conclusions

Coffee powders in different forms of packaging were not significantly different in terms of DBP and DEHP concentrations. Rather, the method for extracting coffee affected the release of plasticizers, particularly DEHP; PEM reported higher DEHP values (6.65 ng/g) than the other two coffee machines. With the levels of DEHP being higher in coffee beverages than in powder, the lack of PAEs could depend on the machine used to make the coffee beverage, rather than their occurrence in powder. Nevertheless, the PAEs levels are below the SMLs and similarly, the indices of risk assessment did not exceed the threshold values for consumption of 1–6 cups of coffee. Despite the different kinds of packaging and types of extraction, coffee did not pose a meaningful risk to consumers in terms of exposure to DBP and DEHP. However, individuals’ choice of coffee machine could help reduce their exposure to PAEs that are mostly derived from other sources.

## Figures and Tables

**Table 1:** Concentrations (ng/g) of DBP and DEHP expressed as median (min-max) values in coffee powder packed using different materials.

Packaging	DBP (ng/g)	DEHP (ng/g)
Multilayer	0.303 (<LOD-2.368)	5.014 (<LOQ-68.843)
Aluminum	0.578 (<LOD-1.736)	4.921 (<LOD-36.516)
Paper pod	0.226 (<LOD-3.331)	3.857 (<LOD-15.993)

**Table 2:** Concentrations (median, ng/g) of DBP and DEHP in coffee beverages obtained using different coffee machines: Professional espresso machine (PEM), Moka pot (MP), Home espresso machine (HEM).

Machine	DBP (ng/g)	DEHP (ng/g)
Professional espresso machine (PEM)	0.07 (<LOD-0.37)	6.65 (2.58–21.32)
Moka pot (MP)	0.11 (<LOD-0.21)	0.78 (<LOD-2.41)
Home espresso machine (HEM)	0.12 (<LOD-0.25)	0.83 (<LOD-2.98)

## Data Availability

All related data and methods are presented in this paper. Additional inquiries should be addressed to the corresponding author.

## References

[1] Zhang Y.J., Guo J.L., Xue J., Bai C.L., Guo Y. (2021). Phthalate Metabolites: Characterization, Toxicities, Global Distribution, and Exposure Assessment. Environ. Pollut..

[2] Hlisníková H., Petrovičová I., Kolena B., Šidlovská M., Sirotkin A. (2021). Effects and Mechanisms of Phthalates’ Action on Neurological Processes and Neural Health: A Literature Review. Pharmacol. Rep..

[3] Ventrice P., Ventrice D., Russo E., de Sarro G. (2013). Phthalates: European Regulation, Chemistry, Pharmacokinetic and Related Toxicity. Environ. Toxicol. Pharmacol..

[4] USEPA (2012). Phthalates Action Plan.

[5] Giuliani A., Zuccarini M., Cichelli A., Khan H., Reale M. (2020). Critical review on the presence of phthalates in food and evidence of their biological impact. Int. J. Environ. Res. Public Health.

[6] Dhavamani J., Beck A.J., Gledhill M., El-Shahawi M.S., Kadi M.W., Ismail I.M.I., Achterberg E.P. (2022). The Effects of Salinity, Temperature, and UV Irradiation on Leaching and Adsorption of Phthalate Esters from Polyethylene in Seawater. Sci. Total Environ..

[7] Cirillo T., Fasano E., Esposito F., Prete E.d., Cocchieri R.A. (2013). Study on the Influence of Temperature, Storage Time and Packaging Type on Di- n -Butylphthalate and Di(2-Ethylhexyl)Phthalate Release into Packed Meals. Food Addit. Contam. Part A.

[8] Bošnir J., Puntarić D., Galić A., Škes I., Dijanić T., Klarić M., Grgić M., Čurković M., Šmit Z. (2007). Migration of Phthalates from Plastic Containers into Soft Drinks and Mineral Water. Food Technol. Biotechnol..

[9] Wang W., Leung A.O.W., Chu L.H., Wong M.H. (2018). Phthalates Contamination in China: Status, Trends and Human Exposure-with an Emphasis on Oral Intake. Environ. Pollut..

[10] Net S., Sempéré R., Delmont A., Paluselli A., Ouddane B. (2015). Occurrence, fate, behavior and ecotoxicological state of phthalates in different environmental matrices. Environ. Sci. Technol..

[11] European Coffee Federation (ECF) (2019). European Coffee Report 2018/2019.

[12] Lanfranchi M., Giannetto C., Dimitrova V. (2016). Evolutionary Aspects of Coffee Consumers’ Buying Habits: Results of a Sample Survey. Bulg. J. Agric. Sci..

[13] (2004). Regulation (EC) No 1935/2004 of the European Parliament and of the Council of 27 October 2004 on Materials and Articles Intended to Come into Contact with Food and Repealing Directives 80/590/EEC and 89/109/EEC.

[14] (2011). Commission Regulation (EU) No 10/2011 of 14 January 2011 on Plastic Materials and Articles Intended to Come into Contact with Food Text with EEA Relevance.

[15] Isci G., Topdas E.F., Dagdemir E., Genis H.E. (2023). Risk Assessment of Oral Exposure to Phthalates from Coffee Samples Marketed in Turkey. J. Food Compos. Anal..

[16] Sakaki J.R., Melough M.M., Provatas A.A., Perkins C., Chun O.K. (2020). Evaluation of Estrogenic Chemicals in Capsule and French Press Coffee Using Ultra-Performance Liquid Chromatography with Tandem Mass Spectrometry. Toxicol. Rep..

[17] de Toni L., Tisato F., Seraglia R., Roverso M., Gandin V., Marzano C., Padrini R., Foresta C. (2017). Phthalates and Heavy Metals as Endocrine Disruptors in Food: A Study on Pre-Packed Coffee Products. Toxicol. Rep..

[18] di Bella G., Potortì A.G., lo Turco V., Saitta M., Dugo G. (2014). Plasticizer Residues by HRGC–MS in Espresso Coffees from Capsules, Pods and Moka Pots. Food Control..

[19] Tsumura Y., Ishimitsu S., Kaihara A., Yoshii K., Nakamura Y., Tonogai Y. (2001). Di(2-Ethylhexyl) Phthalate Contamination of Retail Packed Lunches Caused by PVC Gloves Used in the Preparation of Foods. Food Addit. Contam..

[20] Cirillo T., Fasano E., Esposito F., Montuori P., Amodio Cocchieri R. (2013). Di(2-Ethylhexyl)Phthalate (DEHP) and Di-n-Butylphthalate (DBP) Exposure through Diet in Hospital Patients. Food Chem. Toxicol..

[21] USEPA (2021). Risk Assessment Guidance for Superfund: Volume III-Part A, Process for Conducting Probabilistic Risk Assessment.

[22] Leclercq C., Arcella D., Piccinelli R., Sette S., le Donne C. (2009). The Italian National Food Consumption Survey INRAN-SCAI 2005–06: Main Results in Terms of Food Consumption. Public Health Nutr..

[23] Health Canada (2010). Contaminated Sites Division Federal Contaminated Site Risk Assessment in Canada.

[24] Fasano E., Bono-Blay F., Cirillo T., Montuori P., Lacorte S. (2012). Migration of phthalates, alkylphenols, bisphenol A and di (2-ethylhexyl) adipate from food packaging. Food Control.

[25] Guo Y., Zhang Z., Liu L., Li Y., Ren N., Kannan K. (2012). Occurrence and Profiles of Phthalates in Foodstuffs from China and Their Implications for Human Exposure. J. Agric. Food. Chem..

[26] Caporaso N., Genovese A., Canela M.D., Civitella A., Sacchi R. (2014). Neapolitan Coffee Brew Chemical Analysis in Comparison to Espresso, Moka and American Brews. Food Res. Int..

